# Theory-informed interventions to promote physical activity and reduce sedentary behaviour in rheumatoid arthritis: a critical review of the literature

**DOI:** 10.31138/mjr.31.1.19

**Published:** 2020-03-31

**Authors:** Sally A.M. Fenton, Joan L. Duda, Jet J.C.S. Veldhuijzen van Zanten, George S. Metsios, George D. Kitas

**Affiliations:** 1 School of Sport, Exercise and Rehabilitation Sciences, University of Birmingham, Edgbaston, Birmingham, United Kingdom,; 2 Department of Rheumatology, Russells Hall Hospital, Dudley Group NHS Foundation Trust, Dudley, United Kingdom,; 3 Faculty of Education Health and Wellbeing, Institute of Sport and Human Science, University of Wolverhampton, Wolverhampton, United Kingdom

**Keywords:** rheumatoid arthritis, physical activity, sedentary behaviour, behaviour change, theory

## Abstract

Moderate-intensity physical activity (PA) is recommended for the management of Rheumatoid Arthritis (RA). Recent evidence suggests that reducing sedentary behaviour (promoting ‘sedentary breaks’ and light intensity PA) may also offer potential for improving RA outcomes, independently of the benefits of moderate-intensity PA. Unfortunately, people living with RA engage in very little moderate-intensity PA, and the spend the majority of the day sedentary. Interventions to support PA and sedentary behaviour change in this population are therefore required. Psychological theory can provide a basis for the development and implementation of intervention strategies, and specify the cognitive processes or mechanisms assumed to result in behavioural change. Application of psychological theory to intervention development and evaluation, therefore, permits evaluation of “how things work”, helping to identify optimal intervention strategies, and eliminate ineffective components. In this review, we provide an overview of existing PA and sedentary behaviour change interventions in RA, illustrating the extent to which current interventions have been informed by psychological theories of behaviour change. Recommendations are provided for future interventional research in this domain, serving as a reference point to encourage proper application of behavioural theories into intervention design, implementation and appraisal.

## INTRODUCTION

Experimental evidence consistently documents the health benefits of engagement in moderate-intensity physical activity (PA) (ie, behaviour ≥ 3 metabolic equivalents [METS]) for people living with RA. These benefits include attenuated inflammatory disease activity, reduced joint pain, decreased fatigue, improved physical function and reduced risk of developing cardiovascular and metabolic disease.^1^ Consequently, participation in PA above a moderate-intensity is recommended as a non-pharmacological intervention for the management of RA and its comorbidities.^2^ This can be accrued as part of planned, structured exercise (eg, running, swimming, cycling, gym classes), or as purposeful PA (eg, a brisk walk).^3^

However, the majority of this patient group remain insufficiently active to accrue such positive health benefits and spend a large proportion of their day engaged in sedentary behaviours (waking behaviour ≤1.5 metabolic equivalents and a sitting or reclining posture). Emerging research suggests sedentary behaviour is linked to deleterious outcomes for people living with RA, including increased long-term cardiovascular risk, vascular dysfunction, reduced physical function, and higher RA disease activity.^4–6^ As such, whilst Moderate-intensity PA may represent an important biological stimulus for reducing the burden of disease in RA, the relative dose of sedentary behaviour accrued by individuals may represent an important exposure in itself, with substantial implications for RA outcomes.^7,8^ Together, evidence for the health impacts of moderate-intensity PA and sedentary behaviour in RA underline a requirement for behavioural interventions that can effectively encourage PA and/or reduce sedentary behaviour in this population.^2,9,10^

To date, the majority of existing interventions in this domain have focussed almost exclusively on promoting PA, rather than directly aiming to reduce sedentary behaviour. These interventions have adopted a number (and combination) of approaches to encourage PA participation, including traditional ‘exercise prescription’, patient education, and behavioural counselling concentrated on addressing barriers, and optimising facilitators to PA. Still, while targeted intervention efforts are certainly being made toward encouraging PA in RA, systematic reviews of current interventions indicate variable levels of success.^11–13^ Crucially, where interventions have demonstrated some success, studies have been unable to explicate promising intervention components: that is, it is not clear which aspects of interventions were effective, and “how” interventions may have worked. This information is critical if we are to be able to move towards generalised behavioural strategies that can successfully support PA in RA. Notably, only one intervention has focussed specifically on reducing sedentary behaviour in RA,^14,15^ owing to which we also know very little about what may comprise a successful intervention in this regard.^16^

In facilitating the identification of effective interventions and their constituents, the application of psychological theory in intervention development and evaluation is increasingly advocated. Indeed, theories provide a systematic way of: 1) identifying psychological factors (determinants) that - if targeted through intervention – are hypothesised to lead to behavioural change, and 2) understanding the psychological processes through which such factors may act to encourage behaviour change.^17^ As such, “theory-based interventions” permit a more comprehensive understanding of “how things work”, thus enabling effective interventions (and/or specific components of interventions) to be identified and optimised, and ineffective constituents to be eradicated.

To date, two published systematic reviews have sought to synthesise the evidence regarding the theoretical basis (and related efficacy) of interventions to promote PA among people living with RA.^12,13^ These reviews included studies published before November 2015 and did not incorporate interventions to reduce sedentary behaviour. Moreover, whilst these reviews provided an initial indication of the current landscape of “theory-based” PA interventions in RA, the narrative was restricted to describing results of interventions, rather than discussing methodological limitations with regard to their use of psychological theory in intervention *development, delivery* and *evaluation*.

To address these shortcomings, we present an in-depth, analytical review of the literature concerning “theory-based” interventions to promote PA or reduce sedentary behaviour in RA.^18^ The primary aim of this review, is to provide an update and overview of the literature on both PA *and* sedentary behaviour change interventions in RA, and illustrate the extent to which psychological theory has been successfully incorporated into intervention *development* (eg, identifying a psychological theory and determinants), *delivery* (eg, selecting practical applications) and *evaluation* (testing the psychological “mechanisms of action”). In doing so, we will answer the overarching question, *“to what extent are PA and sedentary behaviour interventions for people with RA theory-based?”*. Existing interventions will be used highlight best practice, as well as underline short comings with regard to current application of psychological theory. Against this backdrop, recommendations to inform a standardised approach to development, delivery and evaluation of theory-based interventions to promote PA or reduce sedentary behaviour in RA will be proposed.

## WHAT IS A THEORY-BASED INTERVENTION?

A theory-based intervention has used a relevant theoretical framework to identify ‘psychological construct(s)’ that are hypothesised to influence the targeted behaviour. These psychological constructs are referred to as *“determinants”,* and - as a precursor to intervention development - empirical or theoretical evidence should assure that these determinants indeed predict the relevant behaviour (eg, PA or sedentary behaviour). Examples of psychological constructs commonly recognised as being relevant to PA/sedentary behaviour change, are *attitude* (eg, theory of planned behaviour),^19,20^ *self-efficacy* (eg, social cognitive theory),^21–23^ and *autonomy* (eg, self-determination theory).^24–26^ The psychological determinants identified are then used to guide intervention development in two ways. First, they represent targeted mechanisms for an intervention - ie, if impacted, they will (theoretically), lead to behavioural change. Second, they offer a basis for choosing specific intervention strategies or behaviour change techniques (Ie, methods of change). That is, intervention techniques/components can be selected on the assumption they will positively impact this determinant.^27^ In the context of PA and sedentary behaviour change, examples of common intervention strategies/methods of change employed to target relevant psychological determinants include goal setting, feedback, problem solving and action planning.^28–30^ Such strategies have been coined ‘Behaviour Change Techniques’ (BCTs).^31^

**Figure 1.**
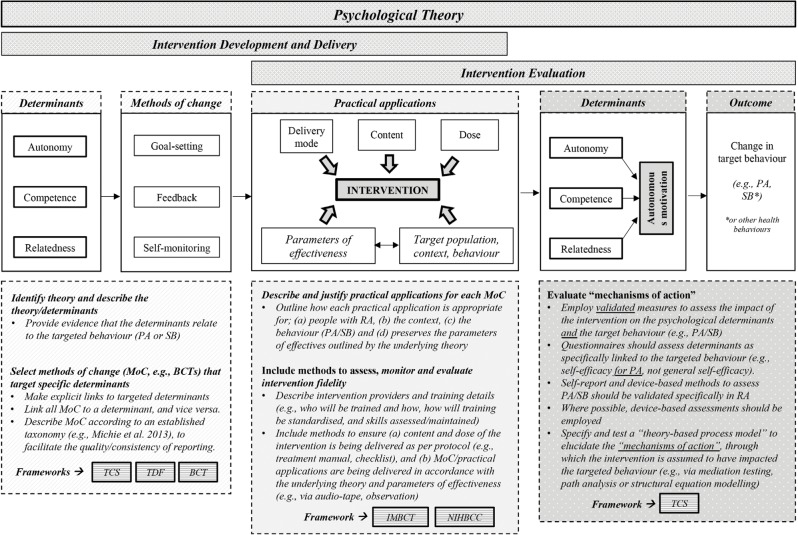
Applying psychological theory to intervention development, delivery and evaluation: steps and recommendations using the example of self-determination theory. *Note:* PA = physical activity; SB = sedentary behaviour; TCS = Theory Coding Scheme^91^; TDF = Theoretical Domains Framework^102^; BCT = Behaviour Change Taxonomy^31^; IMBCT = Intervention Mapping Taxonomy^34^; NIHBCC = National Institutes of Health Behavioural Change Checklist^103^ Dashed boxes represent recommendations for intervention development 
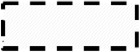
, delivery 
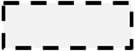
 and evaluation 
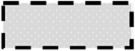
. Guiding frameworks are shown as 
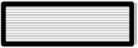

This process of identifying theory-based determinants and matching them with appropriate intervention strategies/methods of change, serves to provide a framework against which hypothesised *“*mechanisms of action*”* can be tested. That is, theory-based *intervention strategies/methods of change* selected for an intervention, can be appraised in terms of their efficacy for influencing the psychological *determinant*, and subsequently, the targeted behaviour. The “mechanisms of action” can be evaluated through constructing and testing a theory-based process model, which examines these successive relationships (ie, *intervention strategies [intervention]* ➔ *determinant* ➔ *behaviour*, see *Figure 1*), and can be conducted using structural equation models, path models and mediation analyses.^32^

By applying psychological theories to inform intervention development and evaluation in this way, we can facilitate understanding regarding specifically *how* an intervention has worked – ie, which psychological constructs/determinants did the intervention successfully target/change, to encourage behavioural change? Specifically, by determining “what works”, this allows effective interventions (or components of interventions/methods of change) to be retained and subsequently generalised, and ineffective interventions to be eliminated and avoided.^33^

### An illustrative example – self-determination theory (SDT)

In *Figure 1*, we use self-determination theory (SDT)^24^ to illustrate the steps required to develop and evaluate a theoretically informed intervention. In brief, SDT posits that social environments which support the needs of autonomy, competence and relatedness, foster a higher quality of motivation (autonomous motivation) towards a behaviour, and lead to more optimal outcomes (eg, increased PA engagement/reduced sedentary behaviour).^24,25^ In this example, the hypothesised SDT-based *determinants* of behaviour to be targeted by an intervention are; autonomy, competence and relatedness. The *intervention strategies/methods of change* (eg, BCTs) selected to target these determinants include (1) goal setting, (2) feedback, and (3) self-monitoring of PA. Following selection of appropriate intervention strategies/methods of change, it is critical to consider how the strategies identified can be translated for practical use – ie, what are the *practical applications,* through which the intervention strategies can be delivered? These practical applications include the mode of delivery (eg, in person, via telephone), as well as the specific content that is delivered, and are combined into an organised programme to form the resulting intervention.^34^ Of vital importance, is that the selection of practical applications is: 1) appropriate for the target population, context and behaviour, and 2) is guided by the theoretical parameters under which the identified intervention strategy/method of change is assumed to be successful – ie, “*the parameters of effectiveness*”.^34^ These parameters are operationalised in the theoretical evidence for a given method, which explicate the specific conditions that must be realised via a practical application, for the chosen method to successfully change behaviour.

Revisiting the example guided by SDT (*Figure 1*), in order to satisfy the parameters of effectiveness, the *practical application* of goal setting requires this intervention strategy to be delivered in a manner that: a) involves the participant to be actively engaged in the goal setting process (ie, they have experienced autonomy), b) ensures the goals set are challenging but achievable (ie, to build competence), and c) in delivering this strategy, care and support for the participant is demonstrated (relatedness). This underlines the importance of making the distinction between simply selecting intervention strategies/methods of change vs. specifically outlining their practical applications to ensure they are aligned with the assumptions of the underlying theory. An intervention which employs several “effective” BCTs (eg, goal setting, modelling, problem solving) may demonstrate limited or no effects if the practical application of these methods do not fulfil the theoretical parameters of effectiveness.^34^ In the sections below, the available literature focussed on PA and sedentary behaviour change interventions in RA is synthesised, and a critical narrative provided regarding the extent to which current interventions are “theory-based” in their approach to *development, delivery* and *evaluation.*

## OVERVIEW OF PHYSICAL ACTIVITY AND SEDENTARY BEHAVIOUR CHANGE INTERVENTIONS IN RA

For this review, we conducted a thorough and extensive literature search to ensure all relevant articles detailing interventions to promote PA or reduce sedentary behaviour were identified. PA was considered to comprise both “lifestyle PA” (accumulated as unstructured/incidental PA, eg, lifestyle-embedded activities [such as housework], incidental movement, slow walking) and/or “structured exercise” (ie, planned, purposeful PA, such as cycling, running, gym/exercise classes, a brisk walk). Sedentary behaviour was operationalised in accordance with the sedentary behaviour research network definition (ie, waking behaviour ≤1.5 metabolic equivalents and a sitting/reclining posture).

### Search criteria and results

**Figure 2.**
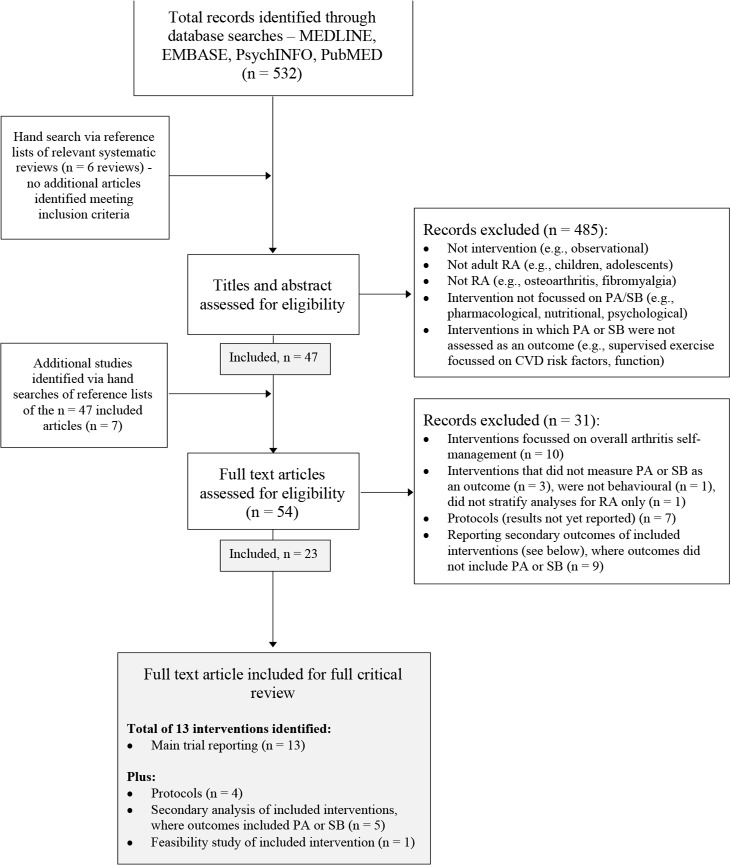
Study selection process. *Note:* PA = physical activity; SB = sedentary behaviour. Full text exclusions: (1) Interventions focussed on overall “arthritis self-management” (to include several topics, eg, medication, pain management, nutrition, problem solving etc.), were excluded to provide a more focussed overview of the current literature (n = 10).^35–44^ ^**^Articles reporting interventions targeting PA/sedentary behaviour and *one* *other behaviour (eg, medication adherence, nutrition) were included, as it was deemed that PA promotion/sedentary behaviour reduction were major components of these interventions;* (2) Interventions where changes in PA or sedentary behaviour were not assessed/reported as an outcome (n = 3, where this could not be determined at the title/abstract screening stage),^45–47^ interventions that were not behavioural (n = 1),^48^ and those targeting RA and another population, where interventions effects on RA could not be isolated (n = 1).^49^ (3) Articles describing protocols for RCTs yet to be completed or reported on (n = 7).^50–56^ (4) Studies reporting secondary results for included interventions, but for which change in PA or sedentary behaviour was not the focus (eg, reporting the economic cost of the intervention) (n = 9).^57–65^ Reference lists of relevant systematic review articles were also searched,^12,13,66–69^ but no additional articles meeting inclusion criteria were identified.

To retrieve relevant articles, systematic searches were run on EMBASE, Ovid MEDLINE(R), PsycINFO, and PubMed from inception to August 2019. Article titles and abstracts were searched using the terms, “rheumatoid arthritis” OR “rheumatology” OR “rheum”* AND “physical activity” OR “exercise” OR “walk”* OR “sedentary behaviour” OR “sedentary” OR “sitting” OR “sit”* AND “intervention” or “randomised controlled trial”. We did not stipulate the mention of ‘theory’ within our inclusion criteria, with the purpose of broadening our search to allow a more comprehensive evaluation of the extent to which theory has been applied to existing interventions. The study selection processes administered following searches is detailed in *Figure 2*.

After cross-referencing and removal of duplicates, titles and abstracts of n = 532 articles were screened. For inclusion in the full-text review, articles were required to describe studies that: (1) report results of an intervention, (2) included adult participants with RA, (3) delivered a behaviour change intervention directed specifically at encouraging PA or sedentary behaviour, (4) reported an outcome related to the change in PA or sedentary behaviour, (5) were published in English. Studies were excluded were: (1) there was no intervention (ie, observational studies), (2) participants were children, adolescents or another patient group (eg, osteoarthritis), (3) interventions were not focussed on PA or sedentary behaviour, (4) PA or sedentary behaviour was not assessed as an outcome. Following screening, n=47 articles were retained for full review, and a further n=7 articles were identified via hand-searching reference lists of included full-text articles.

After full-text review, a further n=31 articles were excluded from the full final critical review.^12,13,35–69^ Reasons for full text exclusions are detailed in *Figure 2*. A total of n=23 articles were included in the final review. Included articles reported the results of thirteen interventions,^15,70–81^ but also included four protocols describing these interventions,^14,81–84^ five articles reporting on secondary outcomes related to PA (ie, long-term follow-ups or theoretical process evaluations)^85–89^ and one feasibility study of an intervention (subsequently delivered as a full-scale Randomised Controlled Trial, RCT).^15,90^

In the sections below, a detailed description of the interventions identified is provided, and a critical narrative regarding the extent to which theory was used to inform intervention development, delivery and evaluation is offered. Specifically, we provide an overview of intervention design, content, delivery and assessments of PA and sedentary behaviour (*Table 1*), detail intervention efficacy (*Table 1*), and appraise the application of psychological theory within these interventions, using a systematic framework – the Theory Coding Scheme (TCS; *Table 2* and *Table 3*).^91^

**Table 1.** Details of interventions promoting physical activity engagement or reducing sedentary behaviour in RA; study aim, design and content, participant characteristics, methodology and results.

**Author and study aim**	**Design and content**	**Participant characteristics** (years, M ± SD) 1. Age, 2. RA duration, 3. Gender (% female)	**Assessment of PA (OB, SR)**	**Outcome and results (PA)**
*Minor et al., 1989* RCT - to evaluate the effects of 12-week exercise programme on exercise tolerance, disease-outcomes and self-reported health status *Minor et al., 1993* – predictors of exercise adherence at 3, 6, and 18 months.	IG^1^ vs. IG^2^ vs. CG n = 40 (group n = not reported) IG^1^= aerobic exercise (walking) IG^2^ = aerobic exercise (aquatics) CG = non-aerobic ROM exercise *Length of intervention:* 12 weeks	1. 54 ± 14 2. 11 ± 8 3. 85%	SR: PA diary. Specific questions/data handling not described. **Not validated in RA.** *Assessments:* baseline, 12 weeks, 6 and 12 months.	**Aerobic exercise (min/week)** - no significant differences between IG and CG at 12 weeks, 6 or 12 months. **Hrs/day ambulating** – IG reported significantly more time in ambulation at 12 weeks compared to CG.
*Brus et al., 1998* RCT - to determine the effects of patient education on compliance (with treatment regimens) and health in patients with RA	IG (n = 25) vs. CG (n = 30) IG = education programme on medication/PA compliance CG = RA brochure *Length of intervention:* 1 year	1. 60 ± 15 (IG) 59 ± 9 (CG) 2. Not reported 3. 80%	SR: Questionnaire - report performance of prescribed physical exercises and endurance activities (walking, swimming, cycling). **Not validated in RA** *Assessments;* baseline, 3, 6, and 12 months.	**Physical exercise (min/week)** – significantly higher in the IG at 3 months compared to the CG. **Endurance activities (min/week)-** no significant difference between IG and CG at any time point.
*Feldthusen et al., 2003* RCT - examining effects of a person-centred physical therapy intervention focussed on health-enhancing PA and balancing life activities on fatigue and fatigue-related variables.	IG (n = 36) vs. CG (n = 34) IG = person centred physiotherapy to tailor PA and balancing life activities. CG = usual care – including visits with rheumatologist and rehabilitation as prescribed. *Length of intervention:* 12 weeks	1. 54 ± 9 (IG) 53 ± 11 (CG) 2. 14 ± 11 (IG) 12 ± 8 (CG) 3. 89%	SR: Leisure Time Physical Activity Index – reported previous 7-day PA. Specific questions/data handling not described. **Not validated in RA.** *Assessments:* baseline, 12 weeks, and 6 months	**MVPA (hours/week) –** significantly greater improvement in MVPA in the IG group compared to the CG, at 12 weeks and 6 months.
*Van den Berg et al., 2006* RCT - to compare the effectiveness of 2 Internet-based PA interventions for patients with RA *Hurkmans et al., 2010 -* 2 year follow-up; n = 110 (56/54)	IG (n = 82) vs. CG (n = 78) IG = internet-based PA programme with individual guidance, bicycle ergometer and group contacts (via website) CG = internet-based programme providing only general information on exercise and PA (via website). *Length of intervention:* 12 months	1. 50 ± 13 (IG) 50 ± 14 (CG) 2. 8 ± 9 (IG) 6 ± 11 (CG) 3. 76%	^**^SR: Questionnaire – report days/week doing; 1) a moderate PA for >=30 minutes in succession, and 2) a vigorous PA for >=20 minutes in succession. **Not validated in RA.** OB: Accelerometer, Actilog 3. Assessed PA in 5-minute epochs over 5 days. 3 days of data used in analysis. **Not validated in RA**. *Assessments;* baseline, 3, 6, 9, 12 and 24 months	**Proportion of participants meeting moderate and vigorous PA recommendations (SR) -** significantly greater in the IG for moderate PA at 6 and 9 months (and vigorous PA at 6, 9 and 12 months), compared to the CG. No significant differences between groups at 2 years. **Average number of accelerations/5-min period/day and total number of 5-min peak activity periods/day (OB)** - no significant differences between IG and CG at any time point.
*Mayoux-benhamou et al., 2008* RCT - to determine the effect of education on the exercise habits of patients with RA at 6 and 12 months	IG (n = 104) vs. CG (n = 104) IG = Multi-disciplinary education programme, including training in home-based exercise and guidelines for leisure time PA. CG = usual care + booklet with home-based exercises and leisure PA recommendations. *Length of intervention:* 12 months	1. 55 ± 12 (IG) 54 ± 14 (CG) 2. 12 ± 10 (IG) 14 ± 10 (CG) 3. 89%	SR: Baecke questionnaire. Specific questions/data handling not described. **Not validated in RA.** *Assessments;* baseline, 6 and 12 months.	**Compliance with leisure time PA (increased score by >=20% from baseline)** – significantly more participants in the IG group complied with leisure PA at 6 months, compared to CG. No significant difference at 12 months. **Overall level of leisure PA** - significantly increased at 6 months only in the IG. Decrease observed in both groups at 12 months.
*Brodin et al, 2008* RCT - to investigate the effect of a 1-year coaching program for healthy PA on perceived health status, body function, and activity limitation in patients with early RA. *Sjoquist et al., 2011 –* 2 year follow-up; n = 228	IG (n = 94) vs. CG (n = 134) IG = coaching programme aimed at implementing healthy PA CG = access to physiotherapy, including education, treatment with physical modalities and organised exercise 2 × week. *Length of intervention:* 1 year	1. 54 ± 14 (IG) 56 ± 14 (CG) 2. 21 ± 5 (IG) 22 ± 4 (CG) 3. 74%	SR: Questionnaire – 3 × questions, regarding frequency of low, moderate and high-intensity PA. Response options; a) never / occasionally, b) 1–3 times/week, c) 4 –5 times/week, and d) 6–7 times/week. **Not validated in RA.** *Assessments*: baseline and 1 year	**Number of participants classified as undertaking ‘healthy PA’ (>=30 mins of moderate or vigorous PA >= 4 days/week)** - no significant differences between IG and CG at 1 year. No significant different at 2 years (Sjoquist et al., 2011).
*Baxter et al., 2015* RCT (feasibility study) - to determine whether an RA walking programme successfully facilitated regular PA, without detriment to pain levels	IG (n = 11) vs. CG (n = 12) IG = Walking intervention – instructions on a walking route, to be completed 3–4 times/week CG = Nutrition education session *Length of intervention:* 6 weeks	1. 67 ± 10 (IG) 59 ± 13 (CG) 2. 9 ± 2 (IG) 6 ± 5 (CG) 3. 80%	OB: Pedometer. Worn for the duration of their involvement in the study. *Assessments:* baseline and 6-weeks.	**Steps/day** – both groups increased their step count at 6 weeks, but there were no significant difference between IG and CG.
*Knittle et al., 2015* RCT - to evaluate the effects of targeting both the motivation and action phases of behaviour change in a 5-week intervention to increase PA among patients with RA not meeting current PA recommendations. *Knittle et al., 2016 –* theoretical process evaluation.	IG (n = 38) vs. CG (n = 40) IG = group-based patient education session + motivational interview (from physiotherapist) + 2 × self-regulation coaching sessions from a rheumatology nurse. CG = group-based patient education session. *Length of intervention:* 5 weeks (with follow-up phone calls focussed on self-regulation for the IG, at weeks 6, 12 and 18).	1. 61 ± 12 (IG) 65 ± 12 (CG) 2. Not reported 3. 67%	^**^SR: 1) Short Questionnaire to Assess Health-Enhancing PA. Report days/week and min/day undertaking walking, cycling and sporting activities; 2) report days/week engaged in >= 30 mins of >=moderate PA in the past month. **Not validated in RA.** *Assessments:* baseline, 6 and 32 weeks.	**Leisure time PA (min/week) –** significant group × time interaction over the 32 weeks of the study. However, no significant difference in change scores from baseline between IG and CG at either 6, or 32 weeks. **Days/week >=30 mins of moderate PA (PA recommendations)** – significant group × time interaction over 32 weeks of the study. A higher % of participants in the IG met the 5 × 30 min/week moderate PA recommendations, compared to the CG, at 6 and 32 weeks.
*Nordgren et al., 2015* Not RCT – to document adherence to and changes in health-enhancing physical activity (HEPA) and functioning, and to explore aspects of adherence and response during the first year of an outsourced 2-year HEPA programme in people with RA. *Nordgren et al., 2018* – 2 year follow-up; n = 117.	All participants received the intervention, n = 220 IG = encouragement for daily PA (pedometer, website), twice weekly circuit training and bi- weekly support group meetings to support PA behaviour change. *Length of intervention:* 2 years	1. 59 ± 9 2. 12 ± 10 3. 81%	SR: International Physical Activity Questionnaire (IPAQ) Short-Form. Categorised as adherers vs non- adherers based on 70% participation in HEPA (5 × 30 mins moderate PA/week). **Low validity for moderate PA in RA.** SR: Exercise Stage Assessment Instrument (ESAI)**,** to determine adherence to HEPA (including muscle strength training >= 2 × week) for > 6 months. *Assessments:* baseline, 1 year, 2 years.	**Current (adherence to) HEPA** - significantly increased from baseline, to 1 year (55% to 82% adherence). Significantly decreased from year 1to year 2 (82% to 75%) (Nordgeren et al., 2018). **Maintained (>6 months) of HEPA** – significantly increased from baseline, to 1 year (0 to 37%). Significantly decreased from year 1, to year 2 (41% to 27%) Nordgeren et al., 2018).
*Garner et al., 2018* RCT (feasibility) - to test the feasibility and effect of a brief individualised counselling intervention on PA levels and fitness, and dietary intake, compared with standard care in RA patients <1 year from diagnosis.	IG (n = 14) vs. CG (n = 14) IG = 2 × individualised nutrition and exercise counselling sessions. CG = usual care *Length of intervention:* 3 months (only 2 sessions).	1. 49 ± 14 (IG) 45 ± 10 (CG) 2. 21 days (IG); 23 days (CG) 3. 82%	OB: Pedometer. Worn for 7 days. *Assessments:* baseline and 6-months.	**Steps/week** - no significant differences between IG and CG.
*Gilbert et al., 2018* RCT - to test the efficacy of the IMPAACT intervention for persons with RA (and OA) in improving arthritis-specific and generic self-reported pain and physical function outcomes, observed measures of function, and objectively measured and self-reported PA levels.	IG (n = 93) vs. CG (n = 92) IG = brief physician recommendation to increase PA to meet national guidelines + motivational interviewing for PA at baseline, 3, 6 and 12-months (year 1, + 2 × sessions in year 2). CG = brief physician recommendation to increase PA to meet national guidelines. *Length of intervention:* 2 years	1. 55 ± 14 (IG) 55 ± 14 (CG) 2. 13 ± 10 (IG) 13 ± 10 (CG) 3. 84%	OB: GT1M Actigraph accelerometer (7 day wear, 60 second epochs, valid wear requirement; >=4 days with >= 10 hours wear/day. MVPA, >=2020 accelerometer counts/min (Troiano et al., 2008). **Cut-points not validated for use in RA** SR: Yale Physical Activity Scale. Report time in PA during a typical week from the past month, and overall estimates of PA over the entire past month. **Some evidence for validity in RA.** *Assessments:* baseline, 3, 6, 12, 18 and 24 months.	**Moderate to vigorous PA (OB)** - no significant differences between IG and CG. **1) total time index; 2) energy expenditure index; 3) activity dimensions summary Index (SR)** - no significant differences between IG and CG.
*Katz et al., 2018* RCT - To test the effect of a pedometer- based intervention on increasing PA and decreasing fatigue among individuals with RA.	IG^1^ (n = 34) vs. IG^2^ (n = 34) vs. CG (28) IG^1^ = education brochure with suggestions of ways to increase PA.+ pedometer + step monitoring diary + step targets (10% above baseline levels, every 2 weeks). IG^2^ = education + pedometer + step monitoring diary CG = education only *Length of intervention:* 20 weeks	1. 55 ± 13 2. 15 ± 12 3. 88%	^**^OB: Pedometer (Jawbone Up). Worn over 7-days. *Assessments;* baseline, 10-weeks (questionnaires only), and 21-weeks.	**Steps/day** - both IGs significantly increased steps/day from baseline, to 21 weeks. The CG significantly decreased steps, from baseline to 21 weeks. Changes within the IGs significantly differed from those in the CG.
**Sedentary behaviour**				
*Thomsen et al., 2017* RCT - to investigate the efficacy of an individually tailored, theory-based motivational counselling intervention on reducing daily sitting time in RA. *Thomsen et al., 2016 –* randomised feasibility study of the same intervention; n = 20	IG (n = 75) vs. CG (n = 75) IG = 3 × motivational interviewing – counselling sessions + individual text message reminders, aimed at reducing daily sitting time. CG = encouraged to maintain usual lifestyle *Length of intervention:* 16 weeks	1. 60 ± 11 (IG) 60 ± 13 (CG) 2. 12 (range, 7 – 20) 3. 69%	^**^OB: ActivPAL 3 ^TM^ activity monitor. Worn over 7 consecutive days. **Validated for use in RA** SR: Physical Activity Scale 2.1 (PAS 2.1). Reported; 1) number of hours/minutes in an average 24 hour day spent sitting at work and during leisure time; 2) the longest continuous time with uninterrupted sitting during work/leisure time. **Not validated for use in RA.** *Assessments;* baseline and 16 weeks,	**Time spent sitting (hours/day, OB** - decreased in the IG (by 1.61 hours/day ) and increased in CG (by 0.59 hours/day), at 16 weeks. Significantly greater difference in change in the IG compared to CG. **Time spent standing/stepping (hours/day, OB)** –daily sitting was replaced by increased standing and stepping. Between-group differences in change of 1.52 and 0.55 hours/day, respectively. **Daily sitting time (at work) –** significant differences in favour of the IG.

Note: IG = intervention group, CG = control group, PA = physical activity, RA = Rheumatoid arthritis; MVPA = moderate-to-vigorous physical activity, SR – self-report, OB = objective.

** indicates PA as study primary outcome.

Values are presented as intervention/control where information for both study arms is provided separately. Where studies included RA + OA participants (Minor et al., 1989, 1993; Knittle et al., 2015), only information for RA subsamples are reported. For participant characteristics (values are presented for the overall sample, or the IG or CG separately where overall aggregates for the sample are not available). Participant characteristics no reported for follow-up studies or theoretical process evaluation as not significant different from baseline assessments.

**Table 2.** Existing interventions in RA: overview of theoretical integration into design and evaluation using the Theory Coding Scheme.

**Autho**r	**1**	**2**	**3**	**4**	**5**	**6**	**7**	**8**	**9**	**10**	**11**	**12**	**13**	**14**	**15**	**16**	**17**	**18**	**19**
Minor et al., 1989; 1993	—	✓	—	—	—	—	—	—	—	—	—	—†	✓A.C	✓^A^	—†	—	✓	—	—
Brus et al., 1998	✓	✓	✓	—	—†	—	—	—	—	—	—	—	—	✓^C^	—	—	—	—	—
Feldthusen et al., 2003	—	—	—	—	—	—	—	—	—	—	—	—†	—	✓^D^	—†	—	—	—	—
Van den Berg et al., 2006; Hurkmans et al., 2010	—	—	—	—	—	—	—	—	—	—	—	—	—	✓^D^	—	—	—	—	—
Mayoux-benhamou et al., 2008	—	—†	—	—	—	—	—	—	—†	—	—	—	—	✓^D^	—	—	—	—	—
Brodin et al., 2008; Sjoquist et al., 2011	✓	—	—	—	—	—	—	—	—	—	—	✓^B^	—†	✓^D^	No	N/A		—	—
Baxter et al., 2015	—	✓	—	—	—	—	—	—	—	—	—	✓^B^	✓ ^A.C,D,E^	✓^B^	No	N/A	—	—	—
Knittle et al., 2015; Knittle et al., 2016	✓	✓	—	—		—	—	✓	N/A	✓	N/A	✓^B^	✓ ^A.C^	✓^C^	✓	✓^D^	Partial	Partial	—
Nordgren et al., 2015; Nordgren et al., 2018	✓	✓	✓	—	—	—	—	—	✓	✓	N/A	✓^B^	✓^B.C,E,F^	—	✓	No	Partial	N/A	—
Garner et al., 2018	—	—	—	—	—	—	—	—	—	—	—	—	✓^E^	✓^C^	—	—	—	—	—
Gilbert et al., 2018	✓	✓	—	—	—	—	—	—		—	—	✓ ^B^	✓^A.C,E,F^	✓^D^	—	—	—	—	—
Katz et al., 2018	—	—	—	—	—	—	—	—	—	—	—	—	—	✓^D^	—	—	—	—	—
**Sedentary behaviour**																			
Thomsen et al., 2017; Thomsen et al., 2016	✓	—†		—	—	—	—	✓	—		—	—†	✓^† E, F^	✓^B^	—†	—	—	—	—

Details of TCS:

[1] Theory/model of behaviour mentioned

[2] Targeted construct (determinant) mentioned as a predictor of the behaviour

[3] Intervention based on a single theory

[4] Theory/predictors used to select recipients for the intervention

[5] Theory/predictors used to select/develop intervention techniques

[6] Theory/predictors used to tailor intervention techniques to recipients

[7] All intervention techniques are explicitly linked to at least one theory—relevant construct/predictor

[8] At least one, but not all, of the intervention techniques are explicitly linked to at least one theory— relevant construct/predictor

[9] Group of techniques are linked to a group of constructs/predictors

[10] All theory—relevant constructs/predictors are explicitly linked to at least one intervention technique.

[11] At least one, but not all, of the theory relevant constructs/predictors are explicitly linked to at least one intervention technique

[12] Theory—relevant constructs/predictors are measured; (a) at least one mentioned in relation to the intervention is measured pre—intervention (b) pre and post intervention

[13] Quality of measures; **theory constructs** — *reliability* = (a) all, (b) at least one (but not all); *validity* = (c) all, (d) at least one (but not all); **behaviour measures** (PA and sedentary behaviour) – (e) evidence for reliability, (f) previously validated.

[14] Randomisation of participants; (a) authors claim randomisation, (b) method of random allocation described, (c) success of randomisation tested (d) randomisation successful

[15] Changes in measured theory—relevant constructs/predictor

[16] Mediational analysis of construct/s/predictors; in addition to 15, (a) Mediator predicts DV? (or change in mediator leads to change in DV), (b) Mediator predicts DV (when controlling for IV), (c) intervention does not predict DV when controlling for mediator, (d) mediated effect statistically significant

[17] Results are discussed in relation to theory. Partial – theoretical constructs discussed, but not tied to overarching theory.

[18] Appropriate support for theory – based on appropriate mediation OR refutation of the theory is based on obtaining appropriate null effects. Partial – support for mediator but not tied to overarching theory.

[19] Results used to refine theory

*Note:* Data extracted according to the Theory Coding Scheme (TCS) criteria for each intervention. Numbers 1—19 refer to TCS criteria (see *Details of TCS*). Symbols indicate TCS criteria was met (= ✓) or not met (= —), and where additional explanation is provided to clarify coding decision (= —†, see Table 3). Where A – F is indicated for items 12–16, this refers to criteria as referred to under *Details of TCS*.

**Table 3.** Existing interventions in RA: detailed description of coding using the TCS.

**Author**	**Theory/model of behaviour mentioned [TCS, 1]; psychological construct mentioned as a predictor of behaviour [TCS, 2]**	**Theory relevant constructs/predictors; used to select/develop intervention techniques and/or tailor techniques to recipients [TCS, 5, 6]; linked to intervention technique(s) [TCS, 7–11]**	**Theory relevant constructs/predictors measured [TCS 12]**	**Changes in theory relevant constructs/predictors measured + evidence for mediation [TCS, 15–16]**
Minor et al., 1989; 1993 *Details related to psychological theory reported in Minor et al., 1993 article.*	Self-efficacy mentioned in relation to exercise behaviour	X	Perceived support for exercise maintenance from family/friends *Measure:* Support for Exercise Scales (SES) **† only measured post-intervention**	**† Changes not assessed** - perceived support from friends for exercise significantly predicted self-directed exercise (min/week) 9 months after the intervention
Brus et al., 1998	Social Cognitive Theory (and self-efficacy mentioned as key predictor of behaviour) *Clearly stated intent to base on Social Cognitive Theory*	Stated original ASMP based on Social Learning Theory, but no details on how theory was used to develop intervention or link theory-relevant constructs to specific techniques	X	N/A
Feldthusen et al., 2003	X	X	Self-efficacy *Measure*: Arthritis Self-Efficacy Scale (ASES) assessed perceived ability to perform specific behaviours to control RA outcomes. **† Self-efficacy not assessed in relation to exercise/PA**	**† Self-efficacy not assessed in relation to exercise/PA.** However, **c**ompared to the CG, trends toward improvements in Arthritis self-efficacy in the IG persisted at follow-up
Van den Berg et al., 2006 Hurkmans et al., 2010	X	X	X	N/A
Mayoux-benhamou et al., 2008	**† Self-efficacy mentioned in relation to health status, not PA**	X	X	N/A
Brodin et al., 2008 Sjoquist et al., 2011	Cognitive Behavioural “Theory” referred to in description of intervention (Brodin et al., 2008)	PA coaches introduced to cognitive behavioural techniques in training. However, psychological constructs that may represent cognitive process underlying PA were not identified or linked to intervention techniques.	Self-efficacy for performing regular PA Outcome expectations for PA **† Measures: Not clear if established/validated measures; 10 point Likert scale used**	No difference in self-efficacy for performing regular PA or outcome expectations for PA between IG and CG at 2 year follow-up (Sjoquist et al., 2011 (not reported for 1 year follow-up, Brodin et al., 2008)
Baxter et al., 2015	Self-efficacy for PA mentioned as a predictor of PA behaviour	X	Self-efficacy (for arthritis symptoms) *Measure:* ASES Self-efficacy (for PA) *Measure:* Self-Efficacy for Physical Activity questionnaire	IG showed moderate improvements in self-efficacy for PA (and ASES), with no changes were observed in the CG. However, change scores were not significantly different between groups
Knittle et al., 2015 Knittle et al., 2016	Self-Regulation Theory (*mentioned – not based on this theory*) Self-efficacy and autonomous motivation for PA mentioned as predictors of PA behaviour	Intervention combined motivational interviewing and self-regulation (SR) coaching to target autonomous motivation and self-efficacy for PA, respectively *BCTs goal setting and feedback during SR coaching mentioned specifically in regard to promoting self-efficacy for PA*	Self-efficacy for PA *Measure:* Self-efficacy to regulate exercise scale Autonomous motivation for PA *Measure:* Treatment self-regulation questionnaire	Significant treatment effects were found for self-efficacy and autonomous motivation (at both 6 weeks and 32-months) Increases in leisure time PA from baseline to 6 weeks, were not mediated by autonomous motivation, self-efficacy or SR skills. However, maintenance of leisure time PA from 6 to 32 weeks, was mediated by greater autonomous motivation. *Partial support for autonomous motivation as psychological mediator*
Nordgren et al., 2015 Nordgren et al., 2018	Social Cognitive Theory *Clearly stated intent to base on this theory* Self-efficacy for exercise, social support for exercise, and outcome expectations in relation to PA, all mentioned as predictors of PA behaviour	Support group meetings – incorporating BCTs (e.g., goal setting, feedback, problem solving) described as an overall approach to target theory-based constructs In addition; 1) goal setting explicitly linked to target self-efficacy and outcome expectations and 2) group format of intervention sessions explicitly linked to social support for exercise	Self-efficacy for exercise *Measure*: Exercise Self- Efficacy Sale Social support for PA from family/friends *Measure*: SES Outcome expectations for PA *Measure:* 2 × questions, “how certain are you that – 1) health enhancing PA is beneficial for your health in the long run?, and 2) has a positive impact on your RA related difficulties?” **†no evidence of validity/reliability for this measure**	At 1 year; 1) social support from friends significant increased, 2) self-efficacy for exercise declined overall, but improved among those adhering more to circuit training or support group meetings Outcome expectations for benefits of PA increased at 2-years. No other changes reported for self-efficacy and social support
Garner et al., 2018	X	X	X	N/A
Gilbert et al., 2018	Self-efficacy (in general) and social support described as being associated with PA behaviour Interaction Model of Client Health Behaviour (IMCHB) *i.e., not a psychological theory per se, but determinants specified within theories of behaviour change were identified and targeted.*	The intervention comprised motivational interviewing, delivered face to face and by telephone (follow-ups). In motivational interviews, Physical Activity Advocates (PAA) employed the Arthritis Comprehensive Treatment Assessment, to systematically assess factors known to influence an individual’s level of physical activity (based on the IMCHB)	Motivation for PA (perceived competence)– authors assessed perceived competence, and incorrectly used these terms interchangeably. *Measure:* Perceived Competence Scale Beliefs related to PA (Cognitive Appraisal) *Measure:* Specifically designed for this study 12 items upon which the patient rates their beliefs about being physically active from “does not describe me at all”, to “describes me exactly” (0–3) Life worries (affective response) *Measure:* Based on the Social Functioning Scale Social support for PA – more specifically, the scale employed assesses autonomy support for PA (different to social support). *Measure:* Health Care Climate Questionnaire (HCCQ)	The effect of the intervention on targeted constructs (perceived competence, beliefs related to PA and life worries) was not reported (Gilbert et al., 2018)
Katz et al., 2018	X	X	X	N/A
**Sedentary behaviour**				
Thomsen et al., 2017 Thomsen et al., 2016	Behavioural Choice Theory *Clearly stated intent to base on this theory (in feasibility paper)* † **General self-efficacy (not specific to reducing sitting time), broadly linked to behaviour change**	Motivational interviewing described as the broad approach used to increase self-efficacy in terms of reducing sitting time Verbal persuasion cited as a BCT used to more explicitly “boost” self-efficacy (*in feasibility study*)	General self-efficacy *Measure:* General Self- Efficacy Scale, assessed a general sense of perceived self-efficacy. † **Self-efficacy not assessed in relation to reducing sitting time)**	Statistically significant differences in favour of the IG were found in general self- efficacy**. † Not self-efficacy for reducing sitting time**

Note:

† cross-referenced with Table 2 where negatively coded criteria required further explanation.

PA = Physical activity; ASES = Arthritis Self-Efficacy Scale; SES = Support for Exercise Scales; IG = intervention group; CG = control group; BCT = Behaviour Change Technique.

### Data extraction

Design: Ten interventions were tested via RCTs, which compared a single intervention group with a control condition (eg, information/advice only, usual care). Only one intervention targeted reductions in sedentary behaviour.^15^ Two articles reported RCTs comparing two different PA intervention groups with a control,^70,81^ and one study delivered a PA intervention to all participants (ie, non-RCT).^78^

Content: One intervention exclusively focussed on delivering supervised, practical exercise sessions to encourage uptake of PA beyond the clinical context. Specifically, Minor et al. compared two different aerobic interventions (walking and swimming), with the aim of improving exercise tolerance, RA disease-outcomes and self-reported health status – including practice of aerobic PA each week.^70^ Two interventions were focussed specifically on the promotion of walking; Baxter and colleagues instructed RA patients with the goal of walking a pre-determined route 3–4 times per week,^75^ and Katz et al. provided participants with pedometers and individualised weekly step targets.^81^

Six interventions were comprised of structured educational or counselling sessions, with the aim of providing support for PA or reducing sedentary behaviour. These interventions included: person-centred meetings/discussions to devise a tailored self-care plan for health enhancing PA,^72^ ‘PA coaching’ (eg, goal setting, problem solving) and the provision of information regarding the benefits of PA in RA,^77^ counselling, assessment and instruction on PA,^79^ and motivational interviewing or self-regulation counselling to encourage PA^77,80^ or reduce sitting time.^15^ The remaining four interventions included both practical sessions in which exercise or lifestyle PA could be practiced under supervision, *plus* educational/counselling strategies (eg, group education sessions), in order to support PA behaviour change outside of the health care setting.^71,73,74,78^ For example, Mayoux-Benhamou and colleagues delivered eight weekly group education sessions; four providing information about RA and its medical management, and four devoted to a PA programme. Sessions included expert lectures on PA guidelines and discussion to enhance positive attitude and beliefs about exercise, tailored advice, and workshops including the practice of home-based exercise and aerobic activities.^74^

Delivery*:* Interventions were delivered over timescales ranging from six weeks to two years. Most interventions were delivered by physiotherapists,^72,76–79^ one in conjunction with internet-based ‘coaching’ for PA.^73^ Two interventions were delivered by rheumatology nurses and/or occupational therapists (trained in motivational interviewing),^15,80^ and one article stated more broadly, their intervention was provided by health professionals.^74^ Three interventions were carried out by research team members,^71,75,81^ and one was delivered by trained exercise instructors.^70^

Assessment of PA or sedentary behaviour: Methods used to assess PA were heterogenous, and included validated questionnaires,^72, 74, 78, 80^ single item questions to determine participation in PA toward recommended levels (typically, 5 × 30 mins moderate-intensity PA/week),^73,76,77^ and study-specific self-report diaries to record frequency of specific physical activities (primarily swimming, cycling and walking).^70,71^ Five interventions employed device-based assessments of PA, such as accelerometers^73,80^ and pedometers,^75,79,81^ with two combining device-based and self-report methods.^73,80^ PA was defined as the primary outcome in three interventions.^73,77,81^ Sitting time was the primary outcome in the sedentary behaviour intervention, and was assessed using the activPAL – an accelerometer with inclinometer function, that is currently considered the gold standard for free-living assessment of sitting time.^16,92,93^

### Intervention efficacy

Eight PA interventions demonstrated significant, but modest increases in self-reported PA at the end of the intervention.^70–74,77,78,81^ However, for those with long-term follow-ups, effects were not maintained at 1 year^74^ or 2 years.^73,78^ Only one study that employed a device-based assessment of PA reported a significant effect of the intervention.^81^ Interestingly, van den Berg et al. observed significant intervention effects for self-reported PA (proportion of participants reporting meeting moderate-to-vigorous PA recommendations), but not on daily PA assessed by accelerometer.^73^ Thomsen et al. reported a significant effect of their intervention on objectively assessed sitting time, observing a decrease in the intervention group by 1.6 hours/day by the end of the intervention.^15^

## APPLICATION OF PSYCHOLOGICAL THEORY TO CURRENT INTERVENTIONS

The TCS comprises 19 items that can be used to reliably describe the extent to which theory has been used to guide intervention development and evaluation, providing a rigorous and systematic means to evaluate the theoretical basis of interventions. For each intervention, we reviewed all relevant publications (ie, protocols, feasibility trails, full-scale RCTs, and reporting secondary outcomes relevant to PA), and extracted data according to TCS criteria (*Table 2*).

### Data extraction and evaluation using the TCS

Considering fundamental TCS criteria ([1] and [2]), coding indicated; [1] six studies (46%) mentioned either, (a) a psychological theory, (b) model of behaviour change in their description or reporting of the intervention; and [2] six studies cited psychological constructs/determinants as predictors of PA or sedentary behaviour.

For TCS criteria [1], *Social Cognitive Theory* (or social learning theory),^71,78^ *Self-regulation Theory*^77^ and *Behavioural Choice Theory*^15^ were described as informing interventions. One study identified the “*Interaction Model of Client Health Behaviour*” (IMCHB) as providing the conceptual basis for intervention.^80^ Another study outlined *Cognitive Behavioural Theory* in describing their intervention.^76^ However, Cognitive Behavioural Theory is not a psychological theory per se, but offers a broad approach outlining how cognitive processes can influence emotional and behavioural actions. With regard to TCS criteria [2], two additional studies (15%) described *self-efficacy for PA and/or exercise* as an antecedent of PA behaviour, but did not tie the construct of self-efficacy to a particular psychological theory (or model) to inform intervention content.

Beyond these basic TCS criteria, data extraction indicated that the extent to which psychological theories were applied to guide intervention development and evaluation were variable (*Table 2*). Owing to such heterogeneity, *Table 3* provides a detailed account of all references to psychological theory (or theory-based psychological constructs/determinants) within all thirteen articles reviewed, which have been mapped on to relevant TCS criteria. The sections below provide a critical narrative to supplement these key points and support our evaluation of the extent to which interventions were “theory-based”.

### Social Cognitive Theory:

In their article, Brus et al. introduced *Social Cognitive Theory,*^22^ to underline how strategies - such as highlighting performance accomplishments and providing vicarious experiences - could foster self-efficacy and encourage exercise engagement.^71^ However, their intervention did not stipulate the use of such strategies (ie, methods of change/BCTs), nor were explicit links made between any intervention components and Social Cognitive Theory constructs, to indicate how they would impact self-efficacy.

Also informed by *Social Cognitive Theory*, Nordgren et al. described outcome expectations, self-efficacy for exercise and self-regulation as fundamental theoretical concepts and important determinants of PA engagement.^78^ In this study, support group meetings were held for participants, in which weekly goal setting and planning for PA were encouraged. It was stated the content of support group sessions were informed by an ‘active behavioural learning approach’, in line with Social Cognitive Theory. However, links between specific strategies delivered during meetings (eg, goal setting) and the aforementioned theoretical constructs (eg, self-efficacy) were not described. Nevertheless, individual psychological constructs (ie, outcome expectations for PA, social support for PA, and self-efficacy for exercise) were assessed to evaluate the extent to which the intervention impacted on these hypothesised determinants. Results demonstrated the intervention to have a positive effect on outcome expectations regarding the benefits of PA at 1-year follow up (2-years from baseline), but no significant effects were observed for any theoretical constructs at intervention end (1 year from baseline).

#### Self-regulation Theory:

In their intervention, Knittle and colleagues pointed out that existing PA interventions typically pay little attention to the motivational aspects of behaviour change, and highlighted self-efficacy and autonomous motivation as two key cognitions which bridge the gap between formation of intentions and uptake of behaviour.^77^ The authors also underlined how self-regulatory techniques – such as goal setting – may help to build self-efficacy, and motivational interviewing may assist with the promotion of more autonomous motivation. Accordingly, this intervention (designed to emphasise “*Self-regulation Theory*”)^94^ combined motivational interviewing with ‘self-regulation coaching’ with the aim of enhancing autonomous motivation and self-efficacy for PA in RA - ie, the targeted psychological constructs. In outlining their intervention, several methods of change/BCTs were described as being encompassed within motivational interviewing and self-regulation coaching (eg, goal setting, feedback and action planning). However, no justification was provided to emphasise how these BCTs were expected to impact either self-efficacy or autonomous motivation for PA. In addition, these constructs were considered in isolation, rather than tied to an overarching psychological theory. That is, identifying a specific theoretical framework within which these determinants are incorporated (and may relate to each other), would have allowed a more detailed examination of the or processes of behavioural change (ie, the “mechanisms of action”). Instead, specific intervention components appeared to be based on general concepts guiding self-regulatory processes (eg, self-reflective implementation of change mechanisms), rather than a well-established behaviour change theory per se (eg, *Social Cognitive Theory,*^22^ *Self-determination Theory*^24^).

Nevertheless, Knittle et al. tested a theory-based process model to evaluate the hypothesised “mechanisms of action”.^85^ Mediation analysis examined the effects of this intervention on PA initiation and maintenance through the hypothesised mediators of autonomous motivation, self-efficacy for PA, and use of self-regulation skills. Results revealed the intervention to lead to enhanced self-efficacy, autonomous motivation and leisure time PA from baseline to 6 weeks. However, these initial increases in leisure time PA were not mediated by changes in the psychological constructs. Analysis of follow-up data revealed long-term maintenance of leisure time PA (from 6 weeks to 32-week follow-up), was mediated by greater autonomous motivation and use of self-regulation skills. In this instance, making more obvious linkages between intervention techniques (eg, specific methods of change/BCTs) and targeted theory-based constructs, would have enabled Knittle and colleagues to eliminate redundant intervention techniques, and identify the most effective strategies to enhance intervention efficacy.

#### Behavioural Choice Theory:

In their intervention to reduce sitting time in RA, Thomsen et al., inferred the application of *Behavioural Choice Theory.*^15,90^ Specifically, their intervention targeted personal factors (ie, self-efficacy), which were assumed to influence a person’s choice of replacing an unhealthy reinforcing behaviour (ie, sitting), with less reinforcing and more healthy alternatives (ie, standing, sedentary breaks). Motivational interviewing was a core component of the intervention, supported with the provision of key behavioural messages and text-messages reminding participants of behavioural goals. However, whilst several important BCTs were at the core of this intervention (eg, goal setting, reviewing behaviour goals, problem solving, social support), the authors did not specify exactly how each technique would impact upon an individual’s self-efficacy. Even if we consider this link to be implicit from the generalised explanation of the intervention, only ‘general self-efficacy’ was assessed, rather than ‘self-efficacy for reducing sitting-time’, specifically. This is incongruent with: (1) the manner in which component methods of change/BCTs were framed (ie, with reference to reducing sitting time), and (2) the underlying assumptions of self-efficacy theory (ie, the parameters of effectiveness), which specify self-efficacy as being a situation specific form of self-confidence. Thus, the accurate appraisal of intervention efficacy is limited from a theoretical perspective. Certainly, whilst general self-efficacy significantly increased in the intervention arm, greater changes may have resulted if a more targeted assessment of self-efficacy for reducing sitting time had been incorporated.

*The Interaction Model of Client Health Behaviour (IMCHB):* Gilbert and colleagues described the “Improving Motivation for Physical Activity in Arthritis Clinical Trial (IMPAACT)” intervention, based on the IMCHB.^80^ The IMCHB describes how background (eg, demographics, current health) and dynamic (eg, cognitive appraisal, affective responses) factors may interact to influence health behaviour, offering a model that provides a realistic and purposeful representation how such factors may influence PA behaviour. However, the IMCHB does not specify theoretical links between psychological constructs assumed to influence behavioural outcomes - ie, hypothesising how one observation can be predicted from another. Thus, whilst this study specified several individual psychological determinants as intervention targets (that are stipulated within the IMCHB), these determinants were not linked to an overarching psychological theory. Still, in outlining the conceptual basis for their intervention, Gilbert et al., described empirical links between some of the psychological (dynamic) factors included the IMCHB and levels of PA engagement, and subsequently underlined how motivational interviewing was used to address these factors (hypothesised determinants).

Psychological constructs targeted were described as “motivation” and “social support” for PA, as well as life-worries (affective responses) and beliefs related to PA (cognitive appraisals). In defining their intervention, the authors reported how motivational interviews comprised several methods of change/BCTs (eg, goal setting, action planning, problem solving, decisional control), but they did not explicate exactly which psychological construct each BCT was intended to impact. Similarly, whilst it was clear that the overarching strategy of motivational interviewing intended to target all psychological constructs identified within the IMCHB, none of these constructs were explicitly linked to a single BCT encompassed within the motivational interviewing approach (eg, goal setting). Instead, “a group of techniques [TCS 9]” (ie, motivational interviewing, including several BCTs), were linked to a group of psychological constructs/predictors (“motivation” and “social support”).

The incongruence between psychological constructs defined in the IMCHB and the manner in which they were conceptualised, and subsequently measured in this study should also be highlighted. The measures used to assess “motivation” and “social support” for PA, should be more correctly described as measuring perceived competence (via the perceived competence scale) and autonomy support for PA (via the Health Care Climate Questionnaire) (*Table 3*). Quite remarkably, whilst the authors emphasised the importance of the targeted psychological constructs (eg, motivation) as being central to encouraging PA behaviour change in RA, the effect of the IMPAACT intervention on these hypothesised psychological mediators has not been reported.

#### Cognitive Behavioural Theory:

In their 1-year coaching programme to promote healthy PA, Brodin et al. cited *Cognitive Behavioural Theory* in the description of their intervention.^76^ In this context, the term “theory” is used to describe an overarching cognitive model which outlines “Cognitive Behavioural Therapy” (CBT) approaches to help individuals learn to evaluate their cognitive processes (eg, meanings, judgements, appraisals) to influence more adaptive behavioural (and emotional) responses. The authors described how experienced psychologists held lectures in Cognitive Behavioural “Theory”, in order to train PA coaches in cognitive behavioural techniques for behaviour change. However, the specific psychological constructs that may represent (or be involved in) key cognitive processes underlying PA behaviour were not identified or highlighted as possible antecedents of PA, and thus, were not described as being targeted by the intervention. As such, this intervention was only (loosely) informed by broad psychological concepts and reasoning underlying adaptive behaviour change (eg, CBT), and was not based on a well-established psychological theory relevant to PA behaviour.

#### Self-efficacy for PA and/or exercise:

Both Minor et al. and Baxter et al. described self-efficacy for PA and/or exercise as a predictors of these behaviours in their studies.^75,86^ However, in each case, the construct of self-efficacy was not tied to an overar-ching theoretical framework (eg, Self-efficacy theory or Social Cognitive Theory), and it was not clear how PA or exercise self-efficacy were targeted by the interventions. Still, Baxter and colleagues assessed changes in self-efficacy (for PA) in response to their walking intervention, reporting moderate changes both self-efficacy for PA and walking (steps/day) among intervention participants. However, the changes observed were not significant, and there were no differences in these outcomes between those in the intervention and control groups. In contrast, Minor et al. did not measure self-efficacy in their intervention, but rather assessed “perceived support for exercise maintenance from family and friends”. As such, empirical evidence outlining the role of self-efficacy as a psychological construct influencing exercise behaviour, may not have been considered in informing the design of this 12-week exercise intervention. Instead, references to self-efficacy may have been a post-hoc consideration.

## SUMMARY OF INTERVENTION LIMITATIONS

In sum, existing interventions to tackle the issues of low PA and high levels of sedentary behaviour in RA have demonstrated some success. However, where interventions are argued to be based on a particular psychological theory, it seems they do not apply the selected theory extensively. Indeed, where interventions are implied to be underpinned by psychological theory, the descriptions provided regarding the behavioural aspects of interventions are generally focussed on basic reporting of intervention strategies/methods of change (eg, BCTs), without making clear connections to psychological constructs/determinants and the underlying theory. That is, there is rarely enough information provided to describe how psychological theory has been used to inform the selection and delivery of intervention components/methods of change, and evaluations seldom considered the psychological “mechanisms of action” (see *Figure 1*). Such limited application of psychological theory restricts the degree to which interventions can be deconstructed to confirm effective methods of change/BCTs, which have successfully impacted on hypothesised theory-based psychological constructs. Thus, current interventions to promote PA or reduce sedentary behaviour in RA, appear to be ‘theory-inspired’, rather than ‘theory-informed’.^95^

In the absence of psychological theory, several of the studies reviewed employed established BCTs, perhaps based on the misinformed assumption these strategies will inevitably result in behavioural change. Indeed, a common misunderstanding is that a BCT can represent a theory in, and of itself (eg, “problem solving theory”) and that simply implementing a BCT infers a theoretical underpinning of an intervention, without a consideration of the psychological processes or “mechanisms of action” hypothesised to underlie behavioural change. Rather, psychological theory should be used to guide the selection of BCTs which can act as intervention strategies/methods of change, based on the notion they will impact upon the psychological determinants identified by the selected theory.

Still, even where appropriate theory-based BCTs are identified, a global issue with the application of BCTs is that it is seldom made clear how they are implemented via intervention (ie, their practical applications). For example, in this instance, Knittle and colleagues^77^ and Thomsen et al.^15^ both outlined the specific BCTs employed in their interventions according to an established BCT taxonomy.^31^ However: 1) the approach in which these BCTs were delivered (and whether/why appropriate for the target population), was not always well described (eg, mode of delivery, specific content delivered), and 2) it was not obvious how practical applications of any given BCT were guided by the parameters of effectiveness akin to the underlying psychological theory (*Figure 1*). As outlined, variability in the practical application of these BCTs may hold important implications for behavioural change. For example, goal setting can be patient-led, or goals can be prescribed without patient input. According to SDT,^24^ these two approaches to goal setting hold different consequences for an individual’s degree of autonomous motivation, and ultimately, behavioural change. Together, such poor descriptions of the steps underpinning the *development* (identifying psychological theory and determinants, selecting methods of change/BCTs that can target determinants), *delivery* (practical applications that preserve the parameters of effectiveness) and *evaluation* (testing “mechanisms of action”) of interventions, makes it inherently difficult to understand why (and how) an intervention did (or did not) work. These details also provide the basis for understanding how an intervention might be refined and developed to improve its efficacy. Considering interventions to promote PA and/or reduce sedentary behaviour in RA, whether the state of the current literature simply reflects little actual (or improper) use of psychological theory in these regards, and/or just poor reporting of interventions is not clear. Still, both issues are significantly limiting advances in our understanding regarding the value of theory-based interventions (and specific theories and their constituent parts) for promoting PA and reducing sedentary behaviour in RA.

For example, consider a “theory-based” intervention that is proven unsuccessful - if the theoretical reasoning and choices underlying intervention development and delivery are not articulated in sufficient detail, it is not possible to know if low intervention efficacy is due to a) apparent lack of value of the selected theory (and/or its specific determinants), or b) poor translation of methods of change/BCTs according to the underlying theoretical assumptions and parameters of effectiveness. Similarly, poor description of intervention strategies/methods of change, and the manner in which they are implemented in accordance with underlying theory, makes it inherently difficult to identify effective techniques and replicate the delivery of such strategies in future work to confirm their efficacy. We must therefore be cognisant of these issues when making evaluations regarding the apparent value of theory in current PA and sedentary behaviour interventions in RA. Indeed, the existing systematic review seeking to discern the efficacy of theory-based interventions to promote PA in RA, did not consider such variability in the level reporting when drawing conclusions.^12,96^ Therefore inferences regarding whether the application of theory to PA/sedentary behaviour interventions in RA may have impacted intervention efficacy, may reflect the fact that psychological theory is not operationalised, implemented and tested sufficiently and accurately, rather than a relative inefficiency of theory-based interventions. With this in mind, a full systematic review of PA and sedentary behaviour interventions in RA is required, in which both the application of psychological theory and the quality of reporting are considered. Whilst this narrative review provides a preliminary indication of the literature in this regard, a rigorous, systematic approach – that employs standardised frameworks for evaluation (such as the Theory Coding Scheme) – will enable firm conclusions to be drawn regarding the efficacy of theory-based interventions to promote PA and/or reduce sedentary behaviour in RA.

Finally, an overarching limitation of existing interventions that should be acknowledged, is a distinct absence of fidelity testing – ie, to evaluate the extent to which interventions are delivered as intended. Some studies reported that individuals delivering the intervention were trained to ensure knowledge of the intervention procedure(s) and/or theoretical foundation, and to acquire skills required to deliver relevant intervention techniques (eg, motivational interviewing).^15,76,77,80^ However, protocols did not outline training provisions, nor did they incorporate measures to ensure the intervention had been provided as planned (eg, direct observation/video analysis of behavioural counselling sessions). To address this limitation, study protocols should provide detailed and precise descriptions of intervention techniques, outline how they should be delivered, and also specify methods to assess and evaluate protocol adherence by those delivering the intervention.^97^

## FUTURE RECOMMENDATIONS

To address the problems outlined herein, more guidance on precise and systematic methods for applying psychological theories to PA and sedentary behaviour change interventions in RA are required. Such approaches will serve to facilitate a shared understanding regarding what constitutes a “theory-based” intervention (and how this can be achieved), whilst simultaneously providing scientific methods for assessing the extent to which interventions are theory-based. To provide direction in this domain, we propose a standardised approach to inform the *development, delivery* and *evaluation* of theory-based interventions to promote PA or reduce sedentary behaviour in RA (*Figure 1*).

The intention is that these recommendations will both direct “proper” use of theory in developing and evaluating interventions, as well as improve completeness and transparency in reporting the use of theory in intervention design. Of paramount importance is ensuring the proposed approach is accessible to researchers and practitioners working outside the domains of behavioural science, and seeking to bridge the gap between clinical and behavioural research. Thus, the recommendations provided draw on several established behaviour change frameworks and taxonomies, which can be easily employed by clinical researchers seeking adopt a rigorous approach to behaviour change science. On the basis of these recommendations, future research priorities in the field should also seek to address the following:

1. Additional research is required to elucidate theories of behaviour change that are particularly relevant to the RA population, test these theories (eg, with experimental and prospective studies), and subsequently apply these theories in practice. Intersectoral approaches to understanding/encouraging behaviour change (eg, focussed on psychological, social and physical environmental factors) are no doubt relevant, and their utility should also be explored, particularly with regard to encouraging sedentary behaviour change in RA.^98^
2. Future investigations should also include exploration of specific determinants of sedentary behaviour in RA, in order to ensure interventions targeting sedentariness are properly informed.^99^ Indeed, factors influencing sedentary behaviour are likely different to those associated with participation in PA,^100^ and there is currently a paucity of evidence in this domain. Research exploring determinants of sedentary behaviour should also include a consideration of possible bi-directional associations between sedentary behaviour with RA outcomes (eg, pain, fatigue), which may represent possible causes, as well as consequences of “too much sitting”.
3. Of particular importance will be to develop interventions that seek to promote PA or reduce sedentary behaviour with the aim of improving the most deleterious RA outcomes. In particular, growing evidence for the association between sedentary behaviour and cardiovascular disease – the leading cause of death among people with RA^102^ - points to the potential value of interventions which aim to reduce sedentary behaviour, and improve cardiovascular health in RA.

## TAKE HOME MESSAGES

Explicit use of psychological theory in designing, delivering and evaluating interventions has several benefits, including identifying relevant strategies and understanding the psychological processes underlying behavioural change. This permits evaluation of “how things work”, helping to identify optimal strategies, and establish their efficacy for promoting behaviour change. Currently, interventions to promote PA or reduce sedentary behaviour in RA demonstrate limited application of psychological theory and/or poor reporting, which restricts the degree to which such interventions can be deconstructed and evaluated to confirm effective intervention strategies, that have successfully impacted on hypothesised theory-based psychological constructs. In the future, particular attention should be directed towards generating evidence to inform the development and evaluation of interventions to reduce sedentary behaviour in RA, adopting the steps outlined herein.
